# Patient engagement in the process of planning and designing outpatient care improvements at the Veterans Administration Health‐care System: findings from an online expert panel

**DOI:** 10.1111/hex.12444

**Published:** 2016-02-23

**Authors:** Dmitry Khodyakov, Susan E. Stockdale, Nina Smith, Marika Booth, Lisa Altman, Lisa V. Rubenstein

**Affiliations:** ^1^ RAND CorporationSanta MonicaCAUSA; ^2^ VISN 22 Veterans Assessment and Improvement PACT Demonstration LaboratoryCenter for the Study of Healthcare InnovationImplementation and Policy (CSHIIP)VA Greater Los Angeles Healthcare System (152)Los AngelesCAUSA; ^3^ Center for Implementation Practice and Research Support (CIPRS)VA Greater Los Angeles Healthcare System (152)Los AngelesCAUSA; ^4^ VA Greater Los Angeles Healthcare System (GLA)Office of Healthcare Transformation and InnovationLos AngelesCAUSA; ^5^ Department of Psychiatry and Biobehavioral SciencesUniversity of CaliforniaLos AngelesCAUSA; ^6^ The David Geffen School of Medicine at University of CaliforniaLos AngelesUSA

**Keywords:** ExpertLens, modified Delphi, online expert panel, patient engagement, quality improvement, VA

## Abstract

**Context:**

There is a strong interest in the Veterans Administration (VA) Health‐care System in promoting patient engagement to improve patient care.

**Methods:**

We solicited expert opinion using an online expert panel system with a modified Delphi structure called ExpertLens^™^. Experts reviewed, rated and discussed eight scenarios, representing four patient engagement roles in designing and improving VA outpatient care (consultant, implementation advisor, equal stakeholder and lead stakeholder) and two VA levels (local and regional). Rating criteria included desirability, feasibility, patient ability, physician/staff acceptance and impact on patient‐centredness and care quality. Data were analysed using the RAND/UCLA Appropriateness Method for determining consensus.

**Findings:**

Experts rated consulting with patients at the local level as the most desirable and feasible patient engagement approach. Engagement at the local level was considered more desirable than engagement at the regional level. Being an equal stakeholder at the local level received the highest ratings on the patient‐centredness and health‐care quality criteria.

**Conclusions:**

Our findings illustrate expert opinion about different approaches to patient engagement and highlight the benefits and challenges posed by each. Although experts rated local consultations with patients on an as‐needed basis as most desirable and feasible, they rated being an equal stakeholder at the local level as having the highest potential impact on patient‐centredness and care quality. This result highlights a perceived discrepancy between what is most desirable and what is potentially most effective, but suggests that routine local engagement of patients as equal stakeholders may be a desirable first step for promoting high‐quality, patient‐centred care.

## Introduction

The term ‘patient‐centred care’ typically refers to medical care focused on the patient and his/her health‐care needs.^1^ Achieving patient‐centred care calls for active patient engagement, often defined as patients working in close partnership with care professionals.^2^ To achieve patient‐centred care, health‐care systems, including the Veterans Health Administration (VA), encourage patients to engage in making decisions about their own care. In addition, these systems often aim to engage patients in improving quality and safety of health‐care services^3^ and in shaping health‐care policy.^4^, ^5^, ^6^, ^7^ For example, patients have been engaged through surveys,^3^ sharing their perspectives during focus groups^8^ and joining patient advisory boards.^9^ Yet few investigations have assessed the desirability, feasibility or impacts of engaging patients more deeply in routine organizational improvement and policy activities.

Research suggests that patient and stakeholder engagement may increase health‐care research and policy relevance to community needs,^10^ shape priorities for improving or re‐designing care delivery,^11^, ^12^ improve care quality and patient safety,^13^, ^14^ reduce disparities in care access and outcomes^15^ and improve opportunities for achieving community and policy impacts.^16^ A recent systematic scoping review of empirical evidence on outcomes of public involvement in health‐care policy, however, did not find conclusive evidence of impacts on care, while acknowledging that engagement may have intrinsic value.^17^ Although we are not aware of a systematic review specifically directed at evaluating patient and public engagement in care planning and design, there is a strong interest in this topic and its relationships to care quality and patient safety at local (e.g. clinic or hospital) and/or regional (e.g. health‐care system) levels.^6^, ^18^ Moreover, existing literature on patient engagement in care planning and design suggests that patient input could inform quality improvement initiatives, but not always in a standardized way;^18^ that there is often a gap between intentions to involve patients and the actual engagement of patients;^19^ and that patient engagement may be most effective in improving information that is made available to patients, facilitating access to care services and improving overall care environment.^20^

In this paper, we use a conceptual model of patient engagement^2^ to explore experts’ opinions about engaging patients in planning and designing outpatient care improvements at the VA. Using an online expert panel methodology, we asked a diverse and purposefully selected national group of experts (*n* = 48) with experience in patient engagement to rate desirability, feasibility, stakeholder acceptance and potential outcomes of eight scenarios describing four patient engagement roles (consultant, implementation advisor, equal stakeholder and lead stakeholder) and two health‐care system levels (local and regional).

## Background

### Project context

The VA is the largest integrated health‐care delivery system in the United States with nearly 1400 sites, organized into 21 Veterans Integrated Service Networks, or VISNs – regional systems of care. Serving 8.3 million Veterans, VA offers a full spectrum of inpatient, outpatient and long‐term care services^21^ and provides a unique opportunity to explore patient engagement at local and regional levels. While advancing the delivery of patient‐centred primary care has been a VA goal over more than a decade, patient engagement is a more recent core organizational principle among VA's strategic goals. Empowering Veterans to improve their well‐being, for example, is highlighted as a core goal in the Blueprint for Excellence, a vision statement that provides guidance for improving the care delivery process.^22^

Every year, VA fields the Survey of Health Experiences of Patients (SHEP) to evaluate patient health‐care experiences, with topics ranging from health‐care access to provider interpersonal skills.^3^ The VA has numerous on‐going initiatives that enlist patients and family members in planning and designing care at local hospital and VISN levels. For example, similar to some Federally Qualified Health Centers (FQHCs)^23^ and integrated not‐for‐profit health‐care providers,^24^ each VA facility has Patient Advisory Councils (PACs) that meet regularly and suggest ways of improving services. Finally, besides engaging individual Veterans and their family members, the VA operates a Patient Advocacy Programme in all facilities. This programme aims to improve the health‐care system to better meet patient expectations and works with governmental agencies at the local, state and federal level and non‐governmental groups (e.g. Veterans service organizations) to obtain services and benefits for eligible Veterans.^25^

Although VA engages patients in a number of ways, there is no consensus on the degree to which patients should be involved in improving the VA health‐care system, nor which health‐care settings or organizational levels are best suited for patient engagement in health system planning. Moreover, despite the availability of patient viewpoints, there is little evidence that Veteran patients or families are systematically involved in routine regional and local decision making around on‐going outpatient care priorities, improvements or policies.

### Theoretical framework

Our project is informed by Carman *et al*.'s patient and family engagement framework,^2^ which suggests that patients play three roles. At the lower end of the engagement continuum, patient input is collected, but patients have no decision‐making authority. Their role is to provide information about their preferences and opinions. Patients can become more involved by serving in an advisory role, for example, by joining patient advisory boards. At the upper end of the engagement continuum, patients engage equally in the decision making by serving in the same roles as other stakeholders on decision‐making bodies. This framework also posits that engagement can occur at three levels across the health‐care system: direct care, organizational decision making at the local level and policy decision making at the regional or national level.

After consulting with VA patient representatives and patient engagement specialists, we modified this framework to better fit the VA context (see Table 1). Specifically, instead of ‘patient involvement’, we conceptualize patients as implementation advisors who directly affect the implementation of care delivery changes or quality improvement initiatives. We added a ‘patient leadership’ role to account for situations prioritizing patients' input over other stakeholders’ perspectives.^26^ Because of the project's focus on care planning and design, we focused only on organizational decision making (e.g. care design and practice improvement) at the local (outpatient medical facility) and regional (VISN) VA levels. Although engaging patients at the direct care level is highly important,^27^ we did not include it because, unlike engagement in design decision making at local and regional levels, a vast literature about engagement in direct care already exists. Furthermore, including it would have increased the burden on panel participants.

**Table 1:** Conceptual framework of patient engagement in the design of VA care

Level of the health‐care system where engagement takes place	Patients' roles			
	Consultant	Implementation advisor	Equal stakeholder	Lead stakeholder
Local‐level care planning and design decision making	Scenario 1. Patients' input on care planning and design decisions at VA outpatient clinics or hospitals is solicited on an as‐needed basis (e.g. through surveys, focus groups, advisory council meetings)	Scenario 2. Patients' input and care preferences affect the way changes in care delivery processes are implemented at VA outpatient clinics or hospitals	Scenario 3. Patients' input on care planning and design decisions at VA outpatient clinics or hospitals is valued equally to the input of other stakeholders	Scenario 4. Patients' input in care planning and design decisions in VA clinics or hospitals is more influential than the input of other stakeholders
Regional‐level care planning and design decision making	Scenario 5. Patients' input on care planning and design decisions in Veterans Integrated Service Networks (VISNs) is solicited on an as‐needed basis	Scenario 6. Patients' care preferences affect the way changes in care delivery processes are implemented at the VISN level	Scenario 7. Patients' input on care planning and design decisions at the VISN level is valued equally to the input of other stakeholders	Scenario 8. Patients' input on care planning and design decisions at the VISN level is more influential than the input of other stakeholders

This table lists patient engagement scenarios rated by the panellists, which are classified based on the level of the health‐care system and the role patients play during the engagement process.

## Methods

We designed an exploratory project to identify areas of agreement and disagreement among experts on four roles and two levels of patient engagement. Each role–level combination represents one patient engagement scenario. We note that by ‘patient engagement’, we refer to the mode of collecting input from patients as well as the way that input is used.

This project addressed the following questions:

1. Which scenarios describing patient engagement at VA are most desirable and why?
2. Which patient engagement scenarios are likely to affect patient‐centredness and quality of VA outpatient care the most and are they desirable and feasible?
3. At what level should patients be engaged and what role should they play in planning and designing VA outpatient care?

### Participants

We used our professional networks and a snowball sampling approach^28^ to recruit a diverse, purposeful sample of US‐based experts on patient engagement. We reached out to individuals who have practical experience in involving patients in outpatient health‐care quality and care design decisions both within and outside of VA, who studied patient engagement or who served as patient representatives themselves. First, we contacted the directors of all VA health services and implementation research centres, as well as consultants and patient engagement researchers outside of the VA with whom we had previously collaborated. We also invited Veterans who volunteered with our local VA facility's PAC. We invited these individuals to participate and/or nominate someone to participate in the expert panel. To ensure adherence to our human subjects review, requiring that only patients with recognized expertise be invited, non‐employee patients were recruited only if they were members of an official VA body, such as the PAC.

Interested individuals were asked to register for the three‐round panel by providing their demographic information. Of 59 registered experts, 48 (81%), who all reported familiarity with the VA context, participated in at least one rating round of this online panel (see below for round descriptions). Rating results described in the paper are based on analysis of the input collected from those experts who provided their final ratings (*n* = 28; 58% of 48 participating experts). We found no statistically significant difference between experts participating in Rounds One and Three except for on gender: men were less likely to participate in Round Three.

### Design

We conducted an online modified Delphi expert panel between 25 August and 2 October 2014. Research suggests that the online format allows for engaging experts while avoiding the expense and inconvenience of travel to a centralized location and coordinating busy schedules.^29^ Based on research demonstrating that anonymity may increase participants' readiness to be more honest and to evaluate the perceived value of each other's arguments, participation in our panel was completely anonymous.^30^

We used ExpertLens^™^ – an online previously evaluated system that combines two rounds of questions with a round of statistical feedback and asynchronous, anonymous discussion.^29^ We chose ExpertLens because it allows for iterative engagement of large, diverse and geographically distributed groups of experts; combining quantitative and qualitative data; and exploring group agreement and disagreement.^30^, ^31^ ExpertLens has been used successfully in studies on different topics, including developing national suicide prevention research goals,^32^, ^33^ identifying definitional features of continuous quality improvement in health care,^29^, ^34^ developing quality and performance indicators/measures for patients with arthritis^35^, ^36^, ^37^ and exploring relevance of ethical principles of community‐engaged research in translational science.^38^

In Round One, experts reviewed and rated eight patient engagement scenarios (see Appendix 1). We chose to use scenarios to generate fruitful discussions among participants, reduce the possibility of providing socially desirable answers and engage participants around potentially difficult topics.^39^, ^40^ Based on our conceptual framework, the first four scenarios described patient engagement at local VA outpatient care facilities; the last four described patient engagement at the VISN (regional) level. Patient engagement at each level was illustrated with four variations of the same example that highlighted differences in patients' roles, which ranged from consultation to patient leadership. The role of consultant was operationalized as soliciting patient input on care planning and design decisions at VA on an as‐needed basis (e.g. through surveys, focus groups, advisory council meetings). The role of implementation advisor was described as engagement in the implementation of changes in care delivery based on patients' input and care preferences. The equal stakeholder role referred to situations where the input of patients is valued equally to the input of other stakeholders, whereas the role of lead stakeholder described situations where patients' input was prioritized.

Participants used 9‐point Likert scales to rate each scenario on six criteria, namely feasibility, patient input, physician/staff acceptance, patient‐centredness, impact on health‐care quality and overall desirability (see Table 2). Overall desirability was a summary criterion capturing the expert's considerations about pursuing implementation of the scenario's approach, including consideration of other five criteria.

**Table 2:** Rating criteria

Feasibility – How feasible is it to implement changes in care delivery processes at VA outpatient clinics or hospitals based on patients' input and care preferences? [1*: very unfeasible; 9: very feasible]*
Patient ability – How likely is it that patients will have the interest and skills necessary for providing input on how changes in care delivery processes should be implemented at VA outpatient clinics or hospitals? *[1: very unlikely; 9: very likely]*
Physician/staff acceptability – How likely is it that VA physicians/staff will accept that the patients' perspectives affect the implementation of changes in care delivery processes at VA outpatient clinics or hospitals? *[1: very unlikely; 9: very likely]*
Patient‐centredness – How likely is it that using patients' input to implement changes in care delivery processes at VA outpatient clinics or hospitals will improve patient‐centredness of VA care? *[1: very unlikely; 9: very likely]*
Health‐care quality – How likely is it that using patients' input to implement changes in care delivery processes at VA outpatient clinics or hospitals will improve care quality? *[1: very unlikely; 9: very likely]*
Overall desirability – Considering all of the issues discussed above, how desirable is it to use patients' input to implement changes in care delivery processes at VA outpatient clinics or hospitals? *[1: very undesirable; 9: very desirable]*

We developed the rating criteria based on the results of a recent study that identified different barriers to widespread patient engagement in practice improvement.^5^ We tailored these descriptions for each scenario to draw participants' attention to differences between scenarios, while keeping the same labels for each criterion. Open‐text boxes after each question allowed participants to explain their ratings and note the factors that most affected their answers.

**Figure 1:**
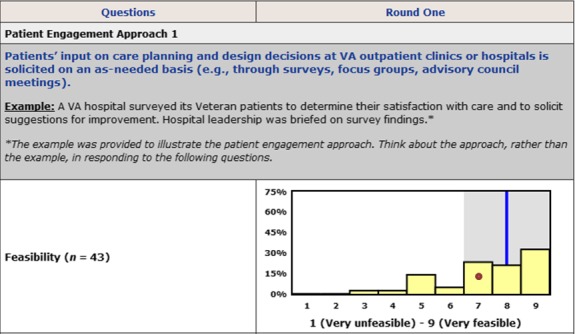
Distribution of Round One answers presented to participants in Round Two. The height of yellow bars is determined by the number of participants choosing a particular response category. A red dot represents a participant's response. A blue line is a group median. A shaded grey area represents an interquartile range.

In Round Two, experts reviewed bar charts showing each participant his/her own response (see Fig. 1) in relation to the distribution of Round One responses. They also interacted with other participants using an anonymous discussion board. Experienced discussion moderators encouraged dialogue by asking participants to explain their perspective on patient engagement, identify barriers and offer suggestions on how these barriers could be overcome.

Finally, in Round Three, participants revised Round One responses in consideration of Round Two feedback and discussion.

RAND Corporation's Human Subjects Protection Committee and the VA Greater Los Angeles Healthcare System's IRB determined this project to be non‐research/quality improvement.

### Data analysis

To determine the final panel decision, we applied the two‐step consensus determination technique described in the RAND/UCLA Appropriateness Method User's Manual^41^, ^42^ to Round Three data. In the first step, we determined the existence of disagreement among participants by calculating the value of the interpercentile range (IPR), or the range of responses between the 70th and the 30th percentiles. We then calculated the value of the interpercentile range adjusted for symmetry (IPRAS), a measure of dispersion for asymmetric distributions, and compared the values of IPR and IPRAS (see Table S1). If IPR>IPRAS, we concluded that disagreement existed among experts, indicating an uncertain group decision due to disagreement. If IPR≤IPRAS, we concluded that there was no disagreement and moved on to step two – determining the group decision. Group decisions could be positive, negative or uncertain without disagreement; they were determined based on median scores. A median score of 7–9 indicated a positive decision (e.g. a scenario was considered desirable, feasible); a median of 1–3 indicated a negative decision (e.g. a scenario was undesirable, not feasible); and a median of 4–6 indicated an uncertain decision without disagreement.

To answer our project questions, we determined group decisions for each question and rank‐ordered patient engagement scenarios on each rating criterion based on the group median responses. We then pooled rank‐ordered data across scenarios at the local and regional levels, regardless of patient role, to determine whether patient engagement at the VISN level is more or less desirable than engagement at the local level. Similarly, we pooled rank‐ordered data across patient roles to explore which role(s) participants consider most desirable, regardless of engagement level.

Finally, we analysed all qualitative data thematically to identify reasons for high or low scenario ratings. We grouped all explanations of numeric responses based on the numeric rating they referred to and linked all discussion board posts to a patient engagement scenario and rating criterion they described. We then coded all text inductively to identify the most prominent themes and those that span across patient engagement scenarios within the same level of engagement or the same patient role. Coded text was reviewed by two team members to ensure coding consistency; disagreements were discussed until consensus was achieved.^34^

## Results

Forty‐five of 48 participating experts (94%) provided Round One ratings; 43 (90%) logged into Round Two; and 28 (58%) provided Round Three ratings. On average, participants logged into the discussion round twice, with some accessing the system up to 10 times. Of 43 Round Two participants, 27 (63%) posted at least one discussion comment. Discussion participants posted 145 comments (mean = 5.37, SD = 3.37; range: 1–14).

Participating experts were predominantly female (77%) and had Master's or higher level of education (85%) (see Table 3). Half reported having research expertise, 31% – clinical expertise, and another 31% – administrative expertise (note that many participants had expertise in multiple areas), while 56% reported working/volunteering at a VA medical facility, 21% reported working/volunteering at the regional or national level. Slightly less than a quarter (23%) reported serving in the armed forces; we considered this to be a proxy for being an actual or potential VA patient.

**Table 3:** Participant demographics (*N* = 48)

Characteristics	%
Gender	
Female	77.1
Education	
AA/some college	6.3
Bachelor's degree	8.3
Master's level education+	85.4
Is a VA employee or volunteer	89.6
Has served in the US Armed Forces	
Yes, has served	22.9
No, has not served	72.9
Missing	4.2
Works in a VA regional/national office	20.8
Has experience working in a clinic (local level)	56.0
(The below categories are not exclusive)	
Is a clinician	31.3
Is a researcher	50
Is an administrator	31.3
Is a volunteer	4.2

### Scenario ratings

Rating data (see Table 4) revealed no significant disagreement among participants; however, none of the patient engagement scenarios received positive median scores (7–9) on all criteria. Two scenarios (S4^1^ and S7) were rated as uncertain (median scores 4–6) on all criteria, and one scenario (S8) received only uncertain or negative ratings (median 1–3).

**Table 4:** Rating results

Patient engagement scenarios	Feasibility		Patient input		Physician/staff acceptance		Patient‐centredness		Health‐care quality		Overall desirability	
	Median	Decision	Median	Decision	Median	Decision	Median	Decision	Median	Decision	Median	Decision
S1. Local level: consultation	8	+	7	+	6	±	7	+	6	±	9	+
S2. Local level: implementation advisor	7	+	7	+	6	±	7.5	+	6	±	8	+
S3. Local level: equal stakeholder	6	±	6	±	5	±	8	+	7	+	7	+
S4. Local level: lead stakeholder	5	±	5	±	4	±	6	±	5	±	5	±
S5. Regional level: Consultation	7	+	6	±	5.5	±	7	+	6	±	7	+
S6. Regional level: implementation advisor	6	±	6	±	6	±	7	+	7	+	7	+
S7. Regional level: equal stakeholder	5	±	5	±	5	±	6	±	5.5	±	6	±
S8. Regional level: lead stakeholder	4	±	5	±	3	−	5	±	4.5	±	5	±

+: A positive decision, meaning that panellists considered a given patient engagement scenario to be feasible, desirable, etc. Shaded cells denote scenarios with positive decisions (a median score of 7–9, without disagreement).

±: An uncertain decision without disagreement, meaning that panellists were uncertain (a median score of 4–6, without disagreement) about the feasibility, desirability, etc, of a given patient engagement scenario.

−: A negative decision, meaning that panellists considered a given patient engagement scenario to be unfeasible, undesirable, etc (a median score of 1–3, without disagreement).

Experts agreed that consulting with patients at the outpatient facility level (S1) is most desirable (median = 9). This scenario also had the highest median values on three other rating criteria, two of which met our standards for a positive group decision. Panellists agreed that soliciting patients' input on care planning and design decisions on an as‐needed basis at VA outpatient clinics or hospitals, which is how S1 was described to participants, was highly feasible (median = 8) and that patients would have interest and skills necessary for providing their input (median = 7). Although this scenario had the highest median rating on the physician/staff acceptance criterion, the median score of six indicated uncertainty among the experts about physicians’ acceptance. Similarly, panellists were uncertain about the impact of local consultations on health‐care quality (median = 6). Experts, however, agreed that these consultations are likely to improve patient‐centredness of VA care (median = 7).

The second most highly rated scenario on the overall desirability criterion described patients as local implementation advisors (S2) (median = 8). This approach had ratings similar to local‐level patient consultation (S1), with two exceptions: its median rating on feasibility was slightly lower (7), and its median rating on patient‐centredness was slightly higher (7.5).

Engaging patients as equal stakeholders at the local level (S3) was also considered desirable (median = 7) and received the highest median rating on the patient‐centredness (median = 8) and health‐care quality criteria (median = 7). However, experts were uncertain about its feasibility (median = 6), the interest and ability of patients to provide input (median = 6) and physician/staff acceptance of patients as equal partners (median = 5).

Patient leadership at the local level (S4) received uncertain ratings on all criteria, with median ratings ranging from 4 to 6.

Similar to S1 (local‐level consultations), panellists’ ratings indicated that regional consultations (S5) were desirable (median = 7) and likely to improve patient‐centredness (median = 7). While experts agreed that it was feasible for patients to be consultants at the regional level (median = 7), they were uncertain about the impact on health‐care quality (median = 6), as well as patient willingness (median = 6) and physician/staff acceptance (median = 5.5).

Experts also agreed that engaging patients as implementation advisors at the regional level (S6) was desirable (median = 7) and likely to have a positive impact on the level of patient‐centredness (median = 7) and health‐care quality (median = 7), but this scenario received uncertain ratings on all other criteria.

S7 describing patients as equal stakeholders at the regional level received uncertain ratings on all criteria, with median ratings ranging from 5 to 6.

Similar to S4 and S7, S8 (patient leadership at the regional level) received uncertain ratings on all criteria with one notable exception: it received a negative rating on physician/staff acceptance (median = 3).

### Scenario rankings

Table 5 contains the desirability rankings for each scenario in relation to all others. Numbers presented in this table are average rankings across all participants for scenarios, with row totals aggregated across patient roles and column totals aggregated across health‐care system levels. A lower mean rank indicates a more desirable response.

**Table 5:** Ranking of patient engagement on the overall desirability criterion

Patients' roles	Level of the health‐care system		Total
	Local	Regional	
Consultant	2.37	4.02	3.20
Implementation advisor	3.04	4.15	3.59
Equal stakeholder	3.58	5.15	4.36
Lead stakeholder	6.40	6.81	6.60
Total	3.85	5.03	

Numbers presented in this table are the average ranks across all participants for scenarios, with totals aggregated across role and health‐care system levels. For example, the mean of 2.37 in the local‐consultant cell could be interpreted as an average rank S1 received across all participants. Numbers in the last column are average ranks of a given patient role, whereas numbers in the last row are the average ranks for all patient engagement roles at each level of the health‐care system. The lower the mean value, the higher a given scenario is ranked on the desirability criterion.

Rankings indicate that local‐level patient engagement was more desirable than regional‐level patient engagement (mean rank of 3.85 across all patient roles at the local level vs. 5.03 at the regional level). They also show that less‐engaged patient roles were rated as more desirable. Nonetheless, although the role of a consultant received the most favourable rank (mean rank of 3.2), participants believed that the roles of implementation advisor and equal partner (mean ranks of 3.59 and 4.36, respectively) would have the highest impact on health‐care quality (data not shown).

### Thematic analysis of participants' comments

In general, participants' comments showed that they had very positive opinions about patient engagement in the design and planning of VA outpatient care. They felt that soliciting patients' input is ‘essential to the survival of the system’. As one expert put it, ‘if you want our Veterans to stay, they MUST [sic] participate. They MUST [sic] feel it is their system. And that way they will be loyal and continue to be a partner in the process of improvement planning’. Several participants noted that ‘patients are the customers’, ‘they are the ones we want to please’ and they are the ones who can identify ‘where glitches in the delivery of care exist’. ‘Without patient input’, noted another expert, ‘care planning becomes one‐sided’.

Although generally positive about patient engagement, discussion comments provided insight into experts’ ratings regarding specific patient engagement approaches. Experts voiced concerns about the feasibility and physician/staff support of even the least intensive approaches to patient engagement (e.g. S1), given the existing organizational structure and culture of VA. As one expert summarized his/her perspective to patients as consultants, ‘there is a potential for feasibility, but currently there are too many barriers for this to happen. The situation is also different depending on the degree of culture transformation at each facility’. Leadership support and resources available for patient engagement were noted as important facilitators.

While some experts felt that it was impossible for patients to be equal partners, others believed that Veteran patients' input should be valued equally to that of other stakeholders. Some indicated that facility or VISN leadership may not be oriented towards patient engagement and argued for a paradigm shift in the organizational culture of VA to consider patients as equal partners in outpatient care planning and design decision making. Similarly, panellists were concerned about physician/staff acceptance of patients as equal partners, citing ‘the level of cynicism directed towards patients'. Some experts argued that patients' input could be as influential as that of other stakeholders only if top leadership insisted on it and if providers and staff were exposed to a collaborative approach to making decisions.

Likewise, experts expressed doubts about patients having a more influential voice than other stakeholders in outpatient care planning and design decisions. Indeed, this role received either uncertain or negative ratings on all criteria at the local and regional levels (see Table 4). Experts reasoned that physicians and staff may not be ready to accept patients as more powerful than other stakeholders. While some experts had reservations about patients' knowledge regarding the logistics of implementing care design decisions or in‐depth understanding of how VA operates, others expressed concern that VA staff and physicians may feel unappreciated, offended and forced to participate ‘in a manner they do not believe in’. Several questioned the representativeness of Veteran patients chosen to voice patients' perspectives and the ability of representatives to remain unbiased.

Discussion comments also provided further insight into the desirability and potential impact of engagement at the local vs. regional levels. As one expert explained, ‘patients may more readily provide input on ways to improve practices at the local level because they have a concrete point of reference that has immediate relevance to them; by contrast, asking for input at the VISN level may feel abstract and less compelling’. The rating data also revealed that three of the five desirable patient engagement scenarios described engagement at the local level. While participants felt that local‐level engagement is desirable, some commented that engagement at the regional level is likely to have a greater impact because ‘more systems are involved [at the VISN level] and [there are] greater implications for action or inaction’. Other experts defended the importance of local engagement, suggesting that engaging patients in local clinics is a building block for engagement at the regional level. ‘Local input is always important and can be channeled to VISN level’, said one expert. ‘Utilizing the local VSOs [Veteran Service Organizations] is always a win’, stated another. ‘This is truly LOCAL [sic] input affecting the LOCAL VHA [sic]. Do this in each VISN supervised hospital to gather consensus info for VISN level action. Win/win’. One strategy of bridging the local–regional divide suggested by experts was to ‘gather [patient] input at the source of care, but topics should include those that will impact VISN policy decisions’.

## Discussion

Our project was designed to explore expert opinion about the desirability, feasibility, stakeholder acceptance and potential outcomes of different roles and levels of engaging VA patients in outpatient care design decision making. Our findings show that experts agreed that soliciting patient input as consultants on care planning and design decisions on an as‐needed basis, including through surveys, focus groups and advisory councils, is currently the most desirable approach to promoting patient influence on VA outpatient care. They also agreed that this approach is feasible, that patients have the interests and skills to contribute as consultants at the local level and that engaging patients this way will have a positive impact on patient‐centredness of VA care.

Our finding about the overall perceived desirability of engaging patients as consultants is consistent with the findings of a recent study on public involvement in health‐care decision making, suggesting that the public generally favours the role of a consultant who provides input, but is not responsible for making the ultimate decision or its consequences.^43^ We hypothesize, based partly on participants' discussion comments and existing literature,^18^ that the overall desirability of this patient engagement approach may be due to its ‘low‐intensity’, relative familiarity to both patients and providers, and Veterans’ willingness to share their care experiences in this manner.

While deemed desirable, the role of a consultant at the local level received uncertain ratings on physician/staff acceptance and health‐care quality. Research conducted in other settings suggests that despite declaring their interest in patient satisfaction surveys, health‐care staff often do not sufficiently discuss survey results, which limits their use in practice and their potential impact on initiating organizational change.^44^ Therefore, it is important to ensure that health‐care professionals recognize the value of patients' experiential knowledge.^20^

Experts also agreed that equal partnership between patients and providers at the local level is most likely to affect the level of patient‐centredness and quality of VA care. Although local equal partnerships did not receive the highest rating on the overall desirability criterion, the positive ratings this approach received on patient‐centredness and care quality suggest that building equal partnerships with patients might be an aspirational goal for VA and other health‐care systems in their attempts to improve patient care. This finding is consistent with a recent recommendation for designing patient engagement approaches that value and give equal weight to contributions of every stakeholder.^18^

Our results also illustrate a contradiction between perceived effectiveness and feasibility of more active patient engagement. Although participating experts generally felt that higher levels of engagement may be more effective, more engaged patient roles were considered to be less feasible. Experts’ uncertainty about the feasibility, patient willingness/interest and physician/staff acceptance of patients as equal stakeholders suggests that health‐care systems interested in patient engagement may need to build patients' capacity and educate physician/staff on how to work in partnership with patients. Recent experimental research on community representation also suggests that it is important to develop on‐going, collaborative and constructive relationships among patients and providers that can change providers’ attitudes towards patient engagement.^45^

In general, patient engagement at the local level received higher desirability and feasibility ratings than patient engagement at the regional level. This finding seems consistent with the result of a UK‐based study showing higher perceived value for engaging patients in practical, operational issues that are likely to generate results in the short term, compared to engaging them in larger‐scale strategic decision making.^20^ Indeed, qualitative data in this project revealed that patients may perceive they have more interest and skills to contribute at the local level. Patients may be more comfortable with the local level because they perceive that their input is not tokenistic, a known concern about patient engagement.^46^ A patient's input might also be easier to apply effectively if it is specific to the place he or she receives care.^47^ Finally, patients may be able to directly observe the impact of their engagement in a facility they visit, and thus experience greater rewards from participating.^48^

Experts’ ratings of patients as local consultants on all criteria were very similar to their ratings of patients as local implementation advisors. However, the latter was rated slightly less feasible and slightly more patient‐centred than the former. The endorsement of more than one patient engagement approach suggests that patients and health‐care systems can choose an approach that they are most comfortable with^18^ or for which they are most ready.

### Limitations

Although innovative and timely, this exploratory project has important limitations. First, as is typical for expert panels,^49^ our participants were not a representative sample of all relevant stakeholders. Therefore, results of this project may be biased towards a more positive view of patient engagement. Second, our final sample consisted of Round Three ratings provided by only 28 experts. Although potentially biased (e.g. it may include only those who liked the online format), this sample is much larger than a sample of nine participants used in traditional expert panels.^41^ Third, this project was focused on the VA. While the observations from this panel process may have relevance to other large national health‐care systems (e.g. the Canadian health‐care system and the United Kingdom's National Health Service) or large managed care organizations (e.g. Kaiser Permanente), the findings may not be representative of other health‐care settings. Fourth, we did not target Veteran patients without patient engagement expertise for participation in the panel. Finally, ExpertLens uses self‐administered surveys, and thus, our results are subject to challenges inherent with this methodology (i.e. reliability, validity).

## Lessons learned

Based on our interpretation of expert opinion collected in this panel, we suggest five lessons learned about patient engagement.

1. Experts agreed that engaging patients as consultants and implementation advisors is relatively feasible, particularly at the local level, and highly desirable. Therefore, VA leaders may want to develop approaches for routinely soliciting patient input on care planning and design decisions.
2. Engaging patients at the local level may be a crucial step towards broader engagement at the regional level because patients are better able to imagine the possible impact and see the actual outcomes of their engagement in their local facility.
3. VA leaders may want to encourage equal partnerships between patients and providers in the process of making care planning and design decisions if the ultimate goal is to improve patient‐centredness and quality of VA care. However, doing so may currently not be feasible, in part because it may require substantial cultural change among physicians and staff.
4. Building patient engagement capacity will require continued effort. Given the engagement barriers, it may be important to educate patients, providers and staff on the benefits of patient engagement; develop best practices and engagement toolkits; and learn how to reward engagement efforts.
5. Health‐care systems may need to provide multiple engagement opportunities so that patients could choose the one that best fits their interests, skills and preferences.

## Supporting information


**Table S1.** IPR and IPRAS Values.

## Note

1 To facilitate the presentation of findings, we used S1 to refer to Scenario 1, S2 to refer to Scenario 2, etc. See Appendix 1 for scenario descriptions.

## Appendix 1 Description of patient engage‐ment scenarios used in the online expert panel

### Scenario 1. Local level: consultation

#### Patients' input on care planning and design decisions at VA outpatient clinics or hospitals is solicited on an as‐needed basis (e.g. through surveys, focus groups, advisory council meetings)

*Example*: A VA hospital surveyed its Veteran patients to determine their satisfaction with care and to solicit suggestions for improvement. Hospital leadership was briefed on survey findings.

### Scenario 2. Local level: implementation advisor

#### Patients' input and care preferences affect the way changes in care delivery processes are implemented at VA outpatient clinics or hospitals

*Example*: A VA hospital surveyed its Veteran patients to determine their satisfaction with care and to solicit suggestions for improvement. Veterans identified the following top three care improvement priorities:

(i) Increase the number of patient parking spaces; (ii) Improve bathroom cleanliness; and (ii) Install check‐in kiosks.

Based on the survey findings and other factors, hospital leadership decided to install check‐in kiosks, the number three priority on the patients' list. Patient representatives were asked to determine the best location for kiosks and helped promote their use among Veterans.

### Scenario 3. Local level: equal partnership

#### Patients' input on care planning and design decisions at VA outpatient clinics or hospitals is valued equally to the input of other stakeholders

*Example*: A local Quality Council, comprising VA providers, administrators and patient representatives, met to prioritize ways to improve care quality in their hospital. While providers felt strongly about focusing on provider continuity (the ability of patients to see their assigned primary care provider), patient representatives insisted on improving the parking situation at the hospital, which was the top priority identified by the patient satisfaction survey (described in Scenario 2). However, after discussing both options, Quality Council members decided not to focus on either one of them. Instead, they agreed to prioritize the installation of check‐in kiosks because both patients and providers felt that check‐in kiosks could improve patients' care experiences.

### Scenario 4. Local level: patient leadership

#### Patients' input in care planning and design decisions in VA clinics or hospitals is more influential than the input of other stakeholders

*Example*: During a previously described local Quality Council meeting convened to prioritize ways to improve care quality in a VA hospital, providers felt strongly about focusing on provider continuity, whereas patient representatives insisted on improving the parking situation. Because parking was the top patient priority identified in the patient satisfaction survey (described in Scenario 2), the Quality Council decided to increase the number of patient parking spaces instead of working to improve continuity.

### Scenario 5. Regional level: consultation

#### Patients' input on care planning and design decisions in Veterans Integrated Service Networks (VISNs) is solicited on an as‐needed basis

*Example*: A VISN located in a predominantly rural area surveyed its patients to determine their satisfaction and solicit care improvement suggestions. VISN leadership was briefed on survey findings.

### Scenario 6. Regional level: implementation advisor

#### Patients' care preferences affect the way changes in care delivery processes are implemented at the VISN level

*Example*: Results of the VISN‐wide patient satisfaction survey identified the following three patient care improvement priorities that were deemed to be equally important by participants:

- Complementary and alternative medicine therapies;
- Telehealth services;
- Improvements in the operation of a regional call centre.

To improve patients' ability to reach clinical services, VISN leadership made a decision to institute performance goals related to timeliness of response by the call centre staff. Veteran representatives were consulted to determine performance targets. Based on their feedback, a 2‐min performance goal was implemented.

### Scenario 7. Regional level: equal partnership

#### Patients' input on care planning and design decisions at the VISN level is valued equally to the input of other stakeholders

*Example*: An analysis of patient survey results (described in Scenario 6) showed Veterans’ interest in using the following to improve anxiety and depression symptoms:

- Hyperbaric oxygen therapy (a treatment in which a patient is placed in a chamber and breathes oxygen at higher than atmospheric pressure)
- Reiki (a form of treatment based on the belief that there is a universal healing energy, the Reiki, which is channelled through the practitioner to the patient to support the patient's body's healing abilities)
- Herbal supplements (plant‐derived compounds used for medicinal purposes)

VISN leadership formed an Advisory Council for Mental Health Services, which included VA providers, administrators and patient representatives, to offer advice on possible introduction of complementary and alternative medicine (CAM) treatment modalities. Although Veteran patient representatives favoured hyperbaric oxygen therapy, providers had strong objections based on the lack of empirical evidence for the use of hyperbaric oxygen therapy to treat anxiety and depression. The Advisory Council, however, agreed to make a recommendation to hire a naturopathic physician and promote the use of herbal supplements.

### Scenario 8. Regional level: patient leadership

#### Patients' input on care planning and design decisions at the VISN level is more influential than the input of other stakeholders

*Example*: In discussing different complementary and alternative medicine (CAM) options for treating anxiety and depression, VA providers on the Advisory Council for Mental Health Services expressed their strong reservations about the effectiveness of different non‐traditional modalities, including Reiki and relaxation therapies. They cited inconclusive evidence of the effectiveness of these treatment modalities and raised concerns about the impact they may have on care quality. Nonetheless, VISN leadership initiated a pilot programme at one outpatient clinic to test Reiki and relaxation therapies based on the overwhelmingly positive patient interest in these treatment options identified by the patient satisfaction survey (described in Scenario 6).
