# Supplementary material for: Patient engagement in the process of planning and designing outpatient care improvements at the Veterans Administration Health‐care System: findings from an online expert panel
Source: Health Expect. 2016 Feb 23;20(1):130–45. doi: 10.1111/hex.12444 (PMC5217877; doi:10.1111/hex.12444)
Supplement: Supplementary file 1 — Table S1. IPR and IPRAS Values. [file HEX-20-130-s001.docx]

**Table: IPR and IPRAS Values**

| **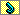Variable** | **IPR** | **IPRAS** |
| --- | --- | --- |
| S1: Feasibility | 2 | 6.85 |
| S1: Patient input | 1 | 6.1 |
| S1: Physician/staff acceptance | 1 | 4.6 |
| S1: Patient-centeredness | 1 | 6.1 |
| S1: Healthcare quality | 1 | 4.6 |
| S1: Overall desirability | 1 | 7.6 |
| S2: Feasibility | 1 | 4.6 |
| S2: Patient input | 1 | 4.6 |
| S2: Physician/staff acceptance | 1 | 4.6 |
| S2: Patient-centeredness | 3 | 6.1 |
| S2: Healthcare quality | 1 | 4.6 |
| S2: Overall desirability | 2 | 6.85 |
| S3: Feasibility | 2 | 3.85 |
| S3: Patient input | 1 | 4.6 |
| S3: Physician/staff acceptance | 1 | 3.1 |
| S3: Patient-centeredness | 2 | 5.35 |
| S3: Healthcare quality | 2 | 5.35 |
| S3: Overall desirability | 2 | 6.85 |
| S4: Feasibility | 2 | 2.35 |
| S4: Patient input | 2 | 2.35 |
| S4: Physician/staff acceptance | 2 | 3.85 |
| S4: Patient-centeredness | 2 | 3.85 |
| S4: Healthcare quality | 1 | 3.1 |
| S4: Overall desirability | 2 | 2.35 |
| S5: Feasibility | 1 | 4.6 |
| S5: Patient input | 2 | 3.85 |
| S5: Physician/staff acceptance | 1 | 3.1 |
| S5: Patient-centeredness | 1 | 4.6 |
| S5: Healthcare quality | 1 | 3.1 |
| S5: Overall desirability | 2 | 5.35 |
| S6: Feasibility | 1 | 4.6 |
| S6: Patient input | 1 | 4.6 |
| S6: Physician/staff acceptance | 2 | 3.85 |
| S6: Patient-centeredness | 1 | 4.6 |
| S6: Healthcare quality | 1 | 4.6 |
| S6: Overall desirability | 0 | 5.35 |
| S7: Feasibility | 2 | 2.35 |
| S7: Patient input | 1 | 3.1 |
| S7: Physician/staff acceptance | 1 | 3.1 |
| S7: Patient-centeredness | 1 | 4.6 |
| S7: Healthcare quality | 2 | 3.85 |
| S7: Overall desirability | 2 | 5.35 |
| S8: Feasibility | 2 | 3.85 |
| S8: Patient input | 1 | 3.1 |
| S8: Physician/staff acceptance | 1 | 4.6 |
| S8: Patient-centeredness | 1 | 3.1 |
| S8: Healthcare quality | 1 | 3.1 |
| S8: Overall desirability | 1 | 3.1 |
